# Macroalgal-Derived Alginate Soil Amendments for Water Retention, Nutrient Release Rate Reduction, and Soil pH Control

**DOI:** 10.3390/gels8090548

**Published:** 2022-08-29

**Authors:** Roelof du Toit van der Merwe, Neill Jurgens Goosen, Robert William McClelland Pott

**Affiliations:** Department of Process Engineering, Banghoek Road, Faculty of Engineering, Stellenbosch University, Stellenbosch 7600, South Africa

**Keywords:** *Ecklonia maxima*, macroalgal-derived alginate, hydrogels, slow-release fertilisers, water retention, reduce nutrient release rate, soil pH control

## Abstract

There is a need to develop sustainably sourced products that can address the needs for improved water retention in soils, slow the release rate of fertilizers (to prevent leaching and downstream eutrophication), and control soil pH for use in agriculture. This article investigates the use of industrial kelp solid waste extracted alginate (IW) slurries to produce soil amendment beads, potentially improving soil water retention, acting as slow-release fertilizers (SRFs), and combined with limestone controls soil pH levels. Alginate extracted from the IW was determined to have a lower guluronic (G) to mannuronic (M) acid ratio than pure laboratory-grade (LG) alginate (0.36 vs. 0.53). Hydrogels produced from the IW alginate achieved significantly higher equilibrium swelling ratios (1 wt% IW = 1.80) than LG hydrogels with similar concentrations (1 wt% LG = 0.61). Hydrogel beads were impregnated with ammonium nitrate and potassium chloride to produce potential SRFs. The release rates of K^+^ and NO_3_^−^ nutrients from the produced SRFs into deionised water were decreased by one order of magnitude compared to pure salts. The nutrient release rates of the IW-based SRFs were shown to be similar to SRFs produced from LG alginate. Hydrogel beads were impregnated with limestone, and it was determined that the alginate-based hydrogels could significantly decrease the nutrient release rate. Using industrial kelp solid waste extracted alginate slurries shows potential for soil amendments production. This report emphasises, for the first time, the use of a crude alginate product in soil amendment formation. Further, it demonstrates slower release rates and soil pH control.

## 1. Introduction

The agricultural sector is facing reduced availability of fertile soil and increasing water scarcity because of changing rainfall patterns due to global climate change [1]. These challenges have led to the need for the development of soil amendments which, firstly, increase water retention within the soil, protecting crops against drought stress [2]. Secondly, soil amendments can assist in soil pH control for optimal crop growth. Thirdly, correctly formulated soil amendments can appropriately supply the required plant nutrients [3].

Most commercial fertilisers are added to the soil as pelletized salts and dissolve relatively quickly during wetting. Fast dissolution rates lead to the leaching of fertilizers into the water table, reducing the longer-term availability of fertilizers for growing plants and causing pollution of surface and sub-surface water, leading to eutrophication of waterways [4,5]. Slow-release fertilisers, designed to release nutrients more gradually, reduce the required fertilizer load and eutrophication potential of fertilisers [6,7]. Other advantages of slow-release fertilisers are that the application frequencies and loading of slow-release fertilisers are lower than conventional fertilisers [8,9].

Soil amendments that counter soil acidity have become crucial to maintaining yields in modern agriculture, as acidity affects the yield and productivity of crops. The pH level below which crops’ growth is restricted differs between crops, but the optimum soil pH for most agricultural cultivated crops is 5.5 to 7.5 [10]. The overuse of fertilisers is one of the main contributors to the acidification of agricultural soils, and the use of ammonium-based fertilisers can significantly increase the acidity of soils. The use of calcium carbonate (limestone) in agriculture has become a common practice to counteract the acidification of soils and increase soil pH [11]. With the addition of calcium carbonate (limestone) to wet soil, the calcium carbonate ionizes, and the carbonate ions react with the cations in the soil, which causes the soil acidity to decrease [12].

The use of absorbent materials in agriculture has attracted attention in recent years since it can increase the water retention properties of soil and help maintain soil moisture during drought stress [13,14]. This is performed by absorbing excess moisture during rainfall events and slowly releasing it over a more extended period than would have occurred otherwise. One class of materials useful in this application is hydrogels, which are hydrophilic polymers capable of absorbing water up to a thousand times their own weight [15,16]. Some studies using hydrogels as soil amendments have been demonstrated in the literature. However, a significant proportion of this research uses synthetic chemical-derived hydrogels composed of, for instance, acrylamide, acrylic acid, glycols and vinyls, which have limitations in terms of large-scale agricultural use because of cost and the non-biodegradability of these hydrogels [17,18,19,20]. Some recent research has focused on the use of bio-composites based on starch acetate, polyvinyl alcohol and glycerol to produce outer hydrophobic coatings for fertilisers in order to decrease the release rates of nutrients [21], while others have experimented with the use of polymer latex films to decrease the release rates of nutrients [22].

Alginate is a natural carbohydrate that can be used to produce biodegradable hydrogels, and in contrast to synthetic polymers, enriches the soil through its degradation [23]. It is a linear co-polymeric carbohydrate with a structural function in brown macroalgal species. Alginate consists of mannuronic (M) and guluronic (G) acid monomers that form either M-block polymers (mannuronic-mannuronic bonds), G-blocks polymers (guluronic-guluronic bonds) or GM-block polymers (guluronic-mannuronic bonds). The ratios and the combinations of these M-, G- and GM-blocks in alginate polymers affect the properties of hydrogels produced from the alginate [24,25]. Macroalgal species rich in alginate (e.g., *Ecklonia maxima*) are used as a source of alginate for industrial application, such as a thickening and gelling agent in the pharmaceutical [26] or food [27] industries. Alginate, once extracted from algal biomass as an alginate salt or alginic acid, can be crosslinked with divalent ions (for instance Ca^2+^), which bind together blocks of guluronic acid forming a hydrogel. These hydrogels can be used for a wide variety of applications [28]. Alginate-based hydrogels exhibit some water retention capabilities and can absorb deionised water up to two times their own weight [29,30,31]. Investigations into the combination of alginate with other polymers to produce copolymer hydrogels have also been performed to potentially increase the swelling capabilities of hydrogels produced from biopolymers [32,33,34].

Previous research into the water absorption and retention capacity of hydrogels in general [35] and alginate-based hydrogels in particular [30,36] show potential for using hydrogels in the field of soil amendments. Some key benefits to using alginate as the hydrogel-forming component, as opposed to other synthetic hydrogels such as polyacrylamide, include that alginate is relatively cheap compared to synthetic polymers, alginate is abundant, and alginate is biodegradable within the soil [37]. The use of seaweed extracts as biostimulants for plant growth has been widely investigated and has shown potential decreasing soil abiotic and biotic stresses [38,39,40]. However, the use of biopolymers is still in an early development stage, and thus most of the life cycle assessment studies that have been done are only based on laboratory-scale datasets [41]. More research needs to be done to ensure that the use of biopolymers in agriculture does not endanger the environment or human health [42].

In addition to their capacity for water retention, hydrogels, such as carboxymethyl cellulose [9], nanocomposites [7], or polyacrylamide [43,44] have been investigated for their use in slow-release fertilizer development. Alginate-based hydrogels also offer this opportunity, and some research has been done in the literature on the development of alginate-based hydrogels (occasionally mixed with other materials) that slowly release, for instance, pesticides [45], biocontrol agents [46], micronutrients [47,48], nitrogen-potassium-phosphorous fertilizers [49], or urea [50,51]. However, there has been little research done on the use of alginate hydrogels formulated for fertilizer release. A potential disadvantage of using purified alginate to produce soil amendment hydrogels for potential usage in agriculture is the relatively high input costs to produce purified alginate compared to pelletized salt fertilisers. In addition, one of the uncertainties in this field that has not been addressed is whether less purified alginate (as a crude extract from the biomass source) will perform equivalently to purified alginate. This investigation reports on this comparison, as the first such work in the literature.

This work demonstrated the production of alginate-based hydrogels, tested as soil amendments, using alginate extracted from locally harvested industrial kelp solid waste [52]. The potential use of these hydrogels as slow-release fertilisers (SRFs) was tested by combining NPK fertilisers with the alginate-based hydrogels. Further, the potential of encapsulating limestone in the produced hydrogels was investigated to determine whether the combination could potentially control soil pH levels. The work is the first to demonstrate a directly useful application of crude-extract alginate, impregnated with various materials, for the improvement of soil conditions.

## 2. Results and Discussion

### 2.1. Production of Alginate-Based Hydrogels

LG alginate-based hydrogel beads were produced using sodium alginate solutions with 1 wt%, 2.5 wt% and 5 wt% sodium alginate. The bead sizes were measured, and only beads between 2.83 mm and 4 mm were used to compare the different hydrogels.

The IW was determined to have a solid content of 51 g/L (±2.4) using the method discussed in Section 4.2.1 and an alginate content of 3.5 g/L (±0.37) using the methods discussed in Section 4.2.2 and Section 4.3.1. Working on the premise of using the industrial waste kelp as a cheap source of alginate, to increase the alginate concentration in the IW, the IW would need to be dewatered, requiring additional energy, which will add extra costs for processing.

Alginate slurries were produced from the IW using the method discussed in Section 4.2.3, and to produce hydrogels with the method discussed in Section 4.2.5, but the extracted alginate slurries were highly viscous and could not be pumped with the use of the peristaltic pumps. Therefore, dilutions were made with the extracted alginate slurries to produce IW hydrogels that would potentially compare to the LG alginate hydrogels produced. Three dilutions were made with the extracted alginate slurries (diluted 1.5, 2 and 5 times with deionised water) to produce industrial kelp solid waste extracted alginate slurries with solid contents of 3.33 wt%, 2.5 wt% and 1 wt%. Hydrogels were successfully produced using the different diluted IW alginate slurries. It is assumed that cellulose and other plant materials in the industrial kelp solid waste significantly contribute to the slurries’ viscosity [53].

### 2.2. Guluronic Acid to Mannuronic Acid Ratio

The guluronic acid (G) and mannuronic acid (M) ratios present in the two alginate sources were investigated since the ratio of these monomers can significantly affect polymer bond strength within hydrogel polymers produced from alginate [54].

Table 1 shows that the LG alginate has a higher G/M ratio when compared to the IW alginate. Alginates with higher concentrations of guluronic acid, in comparison to mannuronic acid, form strong bonds between their polymers and produce more brittle and strong hydrogels [54]. Conversely, alginates with lower G/M ratios produce hydrogels that are often more elastic and slightly weaker compared to hydrogels with higher G/M ratios [55].

The experimentally determined G/M ratios are lower than some literature reported G/M ratios, but the composition of kelp alginate is significantly affected by several factors and thus can be highly variable. The implication for application in soil amendments is that the production process must be sufficiently robust to handle variable compositions of alginate.

### 2.3. Water Retention Tests with Produced Hydrogels

**Figure 1:**
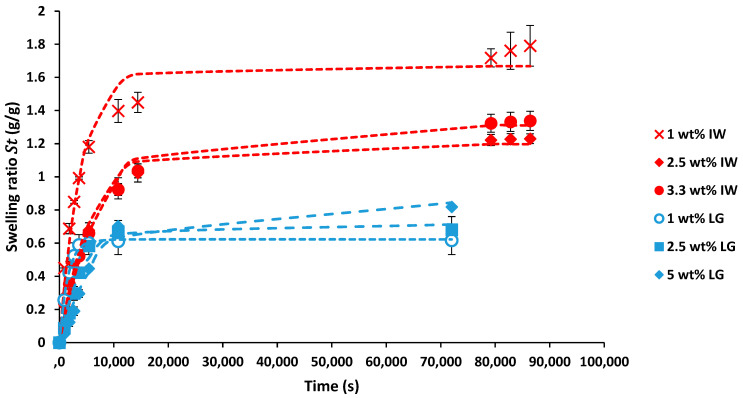
Water retention test results of hydrogels produced using different concentrations (1, 2.5 and 3.3 wt%) of laboratory-grade (LG) alginate solutions and hydrogels produced using industrial kelp solid waste extracted alginate slurries (IW) with different solid contents (1, 2.5 and 5 wt%). The swelling ratio is a fractional indication of the mass of liquid absorbed per mass of dried hydrogel. Error bars show standard deviation from triplicate experimental runs. The dotted lines indicate results achieved through fitting the Voigt model.

Water retention tests were conducted with hydrogels produced from 1, 2.5 and 3.33 wt% solid content IW alginate slurries and hydrogels produced from 1, 2.5 and 5 wt% LG alginate solutions and the results are shown in Figure 1.

The results shown in Figure 1 show that the hydrogels produced using the IW alginate slurries achieved higher swelling ratios when compared to the hydrogels produced using LG alginate solutions. Through ANOVA analysis using a significance level of 0.05, it was determined that there are significant differences between the swelling ratios of the hydrogels produced from the same source of alginate with different alginate concentrations. In addition, for the concentrations of 1 wt% and 2.5 wt%, it was determined that significant differences are observed between the swelling ratios of hydrogels produced from different alginate sources. The swelling ratio measurements were fitted to the Voigt model to determine the rate parameters and the equilibrium swelling ratios of all the hydrogels tested, and the results are shown in Table 2.

From the R^2^ values determined, it is shown that the Voigt model fits the swelling results accurately and can be used to model the swelling of both hydrogels produced from laboratory-grade sodium alginate solutions and hydrogels produced from industrial kelp solid waste alginate slurries. In Table 2, it is shown that the rate parameter (*r*) increases as the alginate concentration in the LG alginate solutions used to produce the LG hydrogels increases. Further, the results in Table 2 show that as the solid content within the industrial kelp solid waste slurries increases, the hydrogels produced rate parameter also increases. This observation is expected because as alginate content within the solution or slurries increases, the amount of G block polymers crosslinked by calcium ions to form the hydrogel network increases. The higher amount of crosslinking within the hydrogel networks causes the hydrogels to become less elastic, and thus the hydrogels cannot swell as much water as hydrogels with lower alginate contents [57,58]. The equilibrium swelling results (*S_e_*) show that the hydrogels produced from the laboratory-grade sodium alginate solutions achieved lower equilibrium swelling when compared to the hydrogels produced from the industrial kelp solid waste slurries. It is assumed that other solids (such as cellulose) within the industrial kelp solid waste slurries affect the hydrogels’ properties and significantly contribute to the swelling capabilities of the hydrogels produced from it.

Previous research reported a rate parameter of 282 s for sodium polyacrylate powders (YanXing SAP) and 228 s for potassium polyacrylate powders (BountiGel-P) [59]. These polyacrylate powders are much faster at absorbing water than the hydrogels produced in this research because of the water retention capabilities of polyacrylate. Work by Rashidzadeh and co-workers [7] used 3 wt% pure sodium alginate solutions to produce sodium alginate-g-poly hydrogels, and they achieved a swelling rate parameter of 2302 s. This result is within the same order of magnitude as the results achieved by the hydrogels produced in this work. From Table 2, the hydrogels produced using the IW alginate slurries have overall higher rate parameters than their LG alginate counterparts. This result indicates that the IW alginate hydrogels are slower at absorbing water to their saturated capacity than the LG alginate hydrogels. This occurrence can be partially attributed to the G/M ratio difference between the different alginate sources and the result that the IW hydrogels have significantly higher swelling capacities than the LG hydrogels. However, other factors such as the actual chain length of the different alginates, other solids within the IW slurries, and seasonal variations can also contribute to the shown differences [60].

### 2.4. Alginate Based Slow-Release Fertilisers

The use of alginate-based hydrogels combined with NPK nutrients was investigated to determine whether slow-release fertilisers could be produced. The potential of producing slow-release fertilisers from alginates extracted from IW was investigated since the production of slow-release fertilisers from IW could potentially be considerably more economical and valuable in the agricultural sector.

#### 2.4.1. Nutrient Concentrations over Time

**Figure 2:**
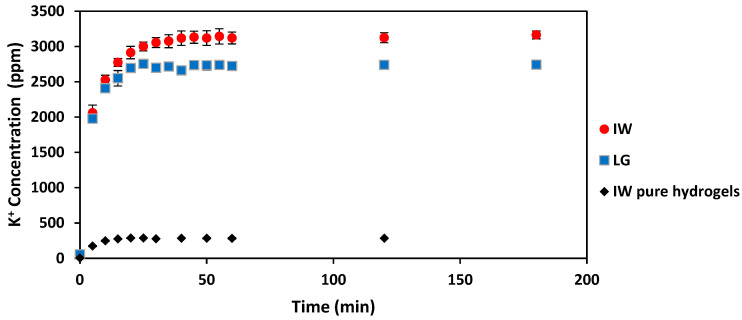
Concentration of K^+^ released during the nutrient release tests for produced slow-release fertilisers (5 wt% alginate in LG and 3.3 wt% IW) over 180 min. The error bars indicate the standard deviation from triplicate experimental runs.

From Figure 2 it is seen that the IW-based slow-release fertilisers released slightly higher concentrations of potassium ions (K^+^) at all the points in time compared to the LG-based slow-release fertilisers’ results. However, the potassium concentrations used to produce the LG-based slow-release fertilisers and the IW-based slow-release fertilisers were the same. Through ANOVA analysis using a significance level of 0.05, it was determined that the differences in steady-state K^+^ concentration between the IW and LG slow-release fertilisers are significant (*p* = 0.0005). The higher steady-state concentrations of K^+^ measured in the IW-based slow-release fertilisers suggest that residual concentrations of K^+^ were present in the IW. Residual concentrations of K^+^ were measured in the IW pure hydrogel (with no added nutrients), as shown in Figure 2.

These results show that the IW slow-release fertilisers can achieve significantly higher equilibrium concentrations of K^+^ in deionised water than the LG slow-release fertilisers. Furthermore, these results indicate that slow-release fertilisers can be produced from the IW that control the release rate of K^+^ just as effective as LG slow-release fertilisers in deionised water at room temperature.

**Figure 3:**
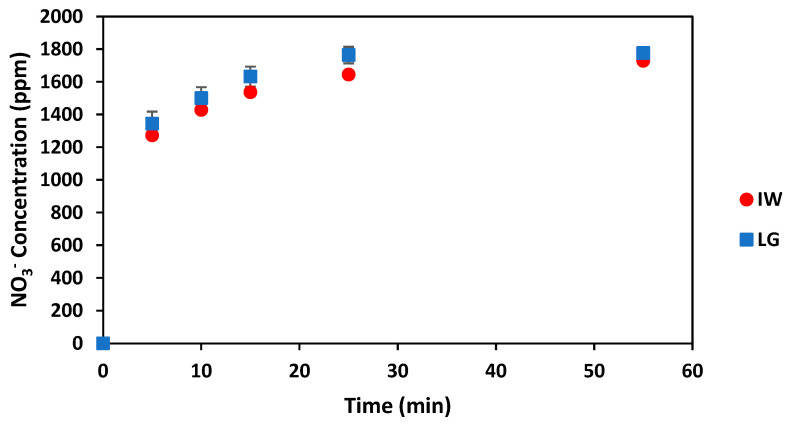
Concentration of NO_3_^−^ released during the nutrient release tests for produced slow-release fertilisers (5 wt% alginate in LG and 3.3 wt% IW) over 55 min. The error bars indicate the standard deviation from triplicate experimental runs.

From Figure 3, the nitrate concentration results are similar for both the produced slow-release fertilisers. The LG-based slow-release fertilisers released a steady-state NO_3_^−^ concentration of 17.76 g·kg^−1^ and the IW-based slow-release fertilisers released a steady-state NO_3_^−^ concentration of 17.28 g·kg^−1^ after 55 min. Statistical analysis confirmed no significant differences between the NO_3_^-^ concentrations released by the produced IW and LG slow-release fertilisers (*p* > 0.05). Thus, the results indicate that the IW-based slow-release fertilisers are just as effective at reducing the nitrate release rate as the LG-based slow-release fertilisers.

The LG-based slow-release fertilisers produced in this research achieved 77.54% total K^+^ released and 94.72% total NO_3_^−^ released after only 55 min in the water, while the IW based slow-release fertilisers from this research achieved 89.05% total K^+^ released and 92.16% total NO_3_^−^ released after only 55 min in water. These release results are relatively fast but are similar to those achieved for releasing potassium and urea from calcium alginate hydrogels modified with yerba mate waste [61]. That work achieved a steady state of nutrient release after 120 min and achieved releases of near 100% for potassium and 72% for urea [61]. The short duration of only 55 min for the steady-state release of the nutrients from the produced slow-release fertilisers can potentially be attributed to the higher mixing rate (500 rpm vs. 300 rpm) that was used during the nutrient release tests for this study compared to the method used by Llive and co-workers [61]. In addition, the sodium alginate used by Llive and others [61] had a higher G/M ratio (1.27) than the alginates investigated in this study which would also affect the nutrient release rates [62]. Copolymers produced from sodium alginate, acrylic acid and acrylamide by Rashidzadeh et al. [7] achieved only 68.3% release of total concentration fertilisers in water after one month. These results are much slower than the nutrient releases achieved in this study, but the nutrient release tests conducted by Rashidzadeh et al. [7] were conducted in stagnant water, and the polymers were produced using synthetic polymer sources.

#### 2.4.2. Diffusivity of the Nutrients from Produced Slow-Release Fertilisers

**Figure 4:**
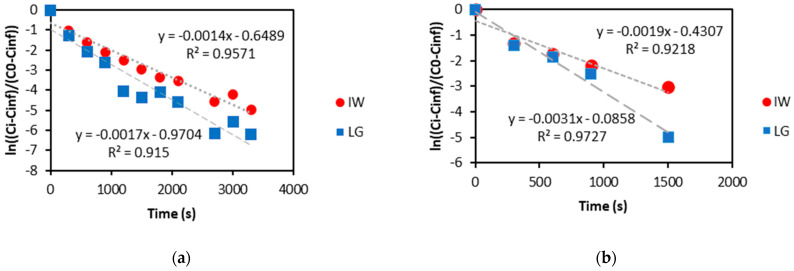
Plot of ln (C_i_ − C_inf_)/(C_0_ − C_inf_) versus time (s) for (**a**) K^+^ and (**b**) NO_3_^−^ nutrient release results for IW and LG slow-release fertilisers to determine the diffusion coefficients using Equation (3).

The diffusion coefficients of K^+^ and NO_3_^−^ ions from the produced slow-release fertilisers into deionised water at room temperature were determined by fitting the nutrient release results to Equation (3). With an increase in the diffusion coefficient, the rate at which the solute diffuses into the solvent increases. The diffusion coefficients were determined using the gradients of the linear trends shown in Figure 4 and Equation (3).

The results in Table 3 show that the diffusion coefficient of K^+^ and NO_3_^−^ from the LG and IW-produced slow-release fertilisers into deionised water are an order of magnitude lower than the diffusion rates of the nutrients at infinite dilutions by Lide et al. [63]. Therefore, suggesting that the LG and IW slow-release fertilisers produced can decrease the diffusion of both potassium and nitrogen into water.

The IW alginate-based slow-release fertilisers had a lower diffusion coefficient than the LG-based slow-release fertilisers and thus are better at decreasing the release rates of K^+^ and NO_3_^−^, but for practical implications, this difference is negligible because they fall within the same order of magnitude. Therefore, it was determined that the IW slow-release fertilisers are just as effective at decreasing the release rate of K^+^ and NO_3_^−^ as the LG-based slow-release fertilisers. Fertilisers are exposed to an active flow of water in soil applications and thus will likely diffuse at higher rates than in passive diffusivity as tested in this experiment. The ions released from the hydrogels would leach into the ground during active flow, decreasing equilibrium effects. It is also expected that the hydrogels would deteriorate over time in the soil, thus thinning the boundary layer, which would increase the release rate of nutrients from the hydrogels.

From Table 3, previous research by Papancea [64] achieved diffusion coefficient results within the same order of magnitude for the diffusion of K^+^ through a PVA membrane into deionised water. Other previous research achieved lower water diffusion coefficients for the diffusion of fertiliser nutrients through a polymer latex film [22]. The lower diffusion coefficients achieved with experiments conducted by An et al. [22] can be attributed to the higher resistance of latex to mass transfer compared to the alginate polymers investigated in this research. The alginate-based hydrogels produced in this research can swell and absorb water, while latex films cannot absorb water. The results show that the slow-release fertilisers produced from the IW are just as effective as the LG alginate-based slow-release fertilisers at reducing the release rate of nutrients. This result further supports the argument of using crude alginate extract, preferably from industrial waste streams, as lower-cost substrates for soil amendment production.

### 2.5. Using Encapsulated Limestone for pH Control

Limestone is widely used in agriculture to control soil pH [65,66]. Ammonium-based fertilisers significantly increase soil acidity, and the use of limestone can counteract this acidification process. The combination of alginate-based hydrogels with limestone was investigated to produce soil amendments that could potentially increase soil water retention and control soil pH levels. Alginate-based hydrogels with added limestone (CaCO_3_) were produced, and the pH altering effects were evaluated in deionised water. The effect of adding limestone and limestone-loaded hydrogels on solution pH, as a proxy for soil pH, was measured with a pH meter to estimate if limestone encapsulated in hydrogels could be released into the water.

**Figure 5:**
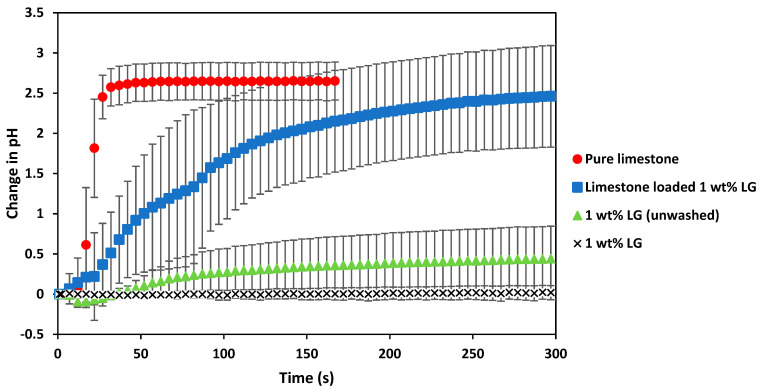
Change in pH over time with the addition of pure limestone (●), 1 wt% laboratory-grade alginate hydrogels (no CaCO_3_ addition) (▲), 1 wt% laboratory-grade alginate hydrogels (no CaCO_3_ addition and washed) (×) and hydrogels with added CaCO_3_ (■). Error bars show the standard deviation between triplicate experimental runs. ANOVA analysis with a significance of 0.05 was applied to the equilibrium results.

In Figure 5, the pH test results of the pure CaCO_3_ dissolved and achieved a steady state after only 70 s. The pure CaCO_3_ changed the pH of the deionised water by 2.65 during this time. These results were expected since the pure CaCO_3_ could quickly dissolve in the deionised water while being stirred to reach its equilibrium. The washed alginate hydrogels had no impact on the pH of the solution, but the unwashed alginate hydrogels caused a slight increase in the pH of the water because of the remaining CaCl_2_ solution within them. However, the differences in these results were determined to be insignificant (*p* > 0.05).

The change in pH observed by adding the alginate limestone hydrogels indicates that the combination of alginate hydrogels with limestone can significantly increase the pH of water over time and achieve similar equilibrium changes in the pH of deionised water as pure limestone. These results confirm that limestone can be encapsulated and released from alginate-based hydrogels. Therefore, these results indicate that adding limestone to alginate hydrogels could potentially be used as soil amendments to control the acidity of soils with the release of limestone. However, more research has to be conducted to investigate the effects of such soil amendments in an acidic soil environment and with the cultivation of crops.

## 3. Conclusions

This study demonstrated for the first time in the literature that alginate hydrogels produced from industrial kelp solid waste extracted alginate slurries could act as effectively as pure laboratory-grade alginate hydrogels in a range of soil amendment applications. In the first instance, these hydrogel beads can quickly adsorb significant quantities of water, recommending them for use in soil water retention. The results further demonstrated significant potential in using industrial kelp solid waste slurries to produce slow-release fertilisers that can reduce potassium ion and nitrate release rates by at least an order of magnitude. However, more research should be carried out on the release rates of the dried produced slow-release fertilisers in soil with active water flow. Further, the addition of limestone to alginate-based hydrogels, as the first demonstration of this method in the literature, proved that limestone could be encapsulated and then released from an alginate-based hydrogel matrix. It is suggested that the combination of fertiliser nutrients with limestone in alginate-based hydrogels should also be investigated in the future to determine whether soil amendments can be produced that can potentially increase soil water retention, control nutrient release, and control soil pH. Further, a cost analysis of the industrial production of industrial kelp solid waste extracted alginate-based slow-release fertilisers would illuminate the potential of this material for more extensive agricultural application.

## 4. Materials and Methods

### 4.1. Materials

The chemicals used in this research includedCaCl_2_, sodium alginate, KCl, HCl, CaCO_3_, NH_4_NO_3_, Ca_3_(PO_4_)_2_ and NaHCO_3_. These chemicals were purchased from Merck (Modderfontein, South Africa) at reagent grade purity (>95%).

For the kelp-derived alginate experiments, wild *Ecklonia maxima* (hereafter referred to as kelp) biomass was harvested from the coast next to Simon’s Town, South Africa. The biomass was initially collected and processed by an industrial partner, Kelpak, who produce biostimulants from the raw kelp. During the industrial processing of the biomass, mature *Ecklonia maxima* plants (including both fronds and stems) were washed with fresh (municipal) water before being shredded and homogenised using a high-pressure homogenizer. The homogenised biomass was then centrifuged to remove most of the liquid, leaving a slurry containing the solid alginate fraction. This slurry (industrial kelp solid waste) was then collected from Kelpak for further processing, as described in Section 4.2.2 below.

### 4.2. Experimental Methods

#### 4.2.1. Solid Content of Industrial Kelp Solid Waste

The insoluble solid content of the industrial kelp solid waste was determined using a gravimetric method where 20 g of wet-based kelp was weighed off and was mixed with 20 mL of deionized water. The sample was thoroughly mixed and then centrifuged at 3000 rpm (2045× *g*) for 5 min to separate the insoluble portion of the biomass from the water-soluble components. The remaining solid material was then again mixed with 20 mL of deionised water, centrifuged again and the supernatant discarded as before. This process was repeated a third time to ensure that all the soluble contents were removed from the samples. The remaining solids in the sample were then dried at 60 °C overnight and weighed. This method was repeated four times to ensure repeatability.

#### 4.2.2. Alginate Content of Industrial Kelp Solid Waste

The alginate content of the industrial kelp solid waste was determined using a method similar to the hydrochloric acid extraction method outlined by Gomez and co-workers [67]. First, 10 g (wet weight) of industrial kelp solid waste, as received from Kelpak^®^, was placed in a 50 mL conical tube and diluted with 40 mL of deionised water. Next, 0.5 g of NaHCO_3_ was added to the solution and mixed on a rotating mixer for 24 h to solubilise the alginate. The solution was then centrifuged at 2770× *g* for 20 min, and the sodium alginate was obtained within the supernatant. Five millilitres of the supernatant was then treated with 1 mL 32% HCl to precipitate the alginic acid. The alginic acid was separated by centrifugation at 2770× *g* for 20 min. Next, the supernatant was eliminated, and the alginic acid precipitate was diluted up to 5 mL with deionised water. Finally, 0.1 g NaHCO_3_ was added to the solution to resuspend the alginate as a sodium alginate solution. The alginate contents of the solutions were determined using the phenol-sulfuric acid method [68], as discussed in Section 4.3.1

#### 4.2.3. Soluble Alginate Produced from Kelp Biomass

In order to prepare the alginate solution from the industrial kelp solid waste, an alkaline dissolution method, as outlined in McHugh [53] and Venkatesan and co-workers [69], was used. Two hundred grams (wet weight) of industrial kelp solid waste as received from Kelpak was placed in a beaker along with 4 g of sodium carbonate. An overhead stirrer stirred the slurry for 24 h to completely dissolve the alginate within the mixture. The resulting slurry was then used for further experimentation without further purification or separation.

#### 4.2.4. Guluronic to Mannuronic Acid Ratio in Alginate

The guluronic to mannuronic (G/M) acid ratios were determined in 1 wt% laboratory-grade (LG) sodium alginate solution and in industrial kelp solid waste extracted (IW) alginate slurries with 1 wt% solids to determine differences between the two sources of alginate. The hydrolysis method described in (Sluiter et al. [70] was used to determine the G/M ratio. First, 72% sulphuric acid was added to a 10 mL sample of 1 wt% alginate solution. After adding the sulphuric acid, the samples were autoclaved at 121 °C for 1 h and then left to cool at room temperature. Next, the samples were cooled down, the pH of the samples was measured, and calcium carbonate salt was slowly added to increase the pH of the samples up to pH 6. The samples were then analysed for guluronic and mannuronic content using HPLC-UV according to the method as used by Wang et al. [71].

#### 4.2.5. Production of Alginate-Based Hydrogel Beads

A sodium alginate solution, dependent on what concentration of alginate hydrogel was to be produced (1, 2, and 5 wt%), was produced by mixing LG sodium alginate powder with deionized water. The mixture was stirred using a magnetic stirrer until the sodium alginate was fully dissolved. A 2 wt% aqueous CaCl^2^ solution was also prepared by weighing off the appropriate salt and dissolving it in deionized water over a stirrer.

The equipment used to produce the hydrogel beads from these solutions is illustrated in Figure 1 below and used a Watson-Marlow 101U/R peristaltic pump (Watson-Marlow Limited, Falmouth, United Kingdom) (2) with peristaltic tubing of 6 mm to control the flow rate and a magnetic stirrer (6)

**Figure 6:**
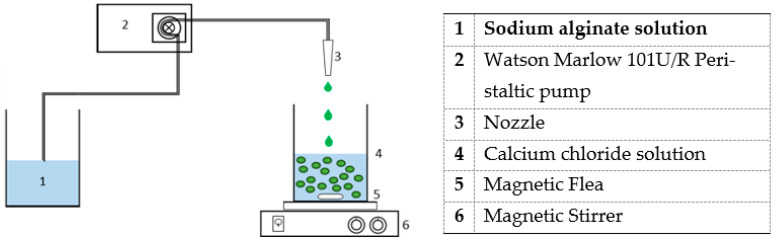
Schematic diagram illustrating the method used to produce alginate-based hydrogel beads.

To form the hydrogels, the alginate solution (1) was pumped at 3.33 mL/s using (2), through a nozzle (3) of 1 mm diameter, which was used to control hydrogel bead size, into the 2 wt% CaCl_2_ solution (4), as shown in Figure 6. These hydrogel beads were left in the calcium solution for 24 h until fully hardened.

The hydrogel beads were then sized, by shaking gently on a set of Taeuber & Corssen (Tauber and Corssen (Pty) Ltd., Johannesburg, South Africa) and Protea (Protea Chemicals, Germiston, South Africa) Test Sieves, to choose a bead size within the size range of <4000 µm diameter, and >2800 µm diameter.

After the hydrogel beads were fully hardened by crosslinking and sized, they were stirred in a beaker overnight with excess deionised water to remove any remaining CaCl_2_ solution. Finally, the hydrogel beads were dried in an oven at 60 °C for 24 h to remove the residual bound water.

#### 4.2.6. Water Uptake and Retention Tests

Swelling tests were conducted with the same method as used in previous research [72,73] to determine the rate of water uptake and the swelling capacity of the hydrogel beads. In these tests, a measured amount of dried hydrogel beads was submerged in deionised water and mixed on a magnetic stirrer at 500 rpm. At regular time intervals, all the hydrogel beads were sieved out of the water, and the outer surfaces of the beads were lightly dried with paper towels to remove excess water. The hydrogel beads were then weighed, the weight change noted, and the hydrogels were returned to the deionized water. The measurements were conducted until an equilibrium weight was reached. The swelling ratio *S_t_* (g/g) at time *t* was calculated using Equation (1), where *W_s_* is the weight of the swollen hydrogel beads, and *W_d_* is the initial weight of the dry hydrogel beads.

(1) $$S_{t}=\frac{W_{s}-W_{d}}{W_{d}\;}$$

The hydrogels’ swelling rate and equilibrium swelling were determined by plotting the swelling ratio results over time and then fitting the Voigt-based viscoelastic model to the results. The Voigt model, as seen in Equation (2), is a spring and dashpot viscoelastic model that has previously been used to describe the swelling rate of superabsorbent polymers [59,74].

(2) $$S_{t}=S_{e}\left(1-e^{-\frac{t}{r}}\right)\;$$

In Equation (2), $S_{t}$ is the swelling capacity at time *t*, *S_e_* is the swelling at steady-state, and r is the rate parameter, also known as the time it takes the hydrogels to achieve 0.63 of the equilibrium swelling. The significance of the regression was also checked between the results measured and those achieved through the Voigt model.

#### 4.2.7. Production of LG Alginate-Based Slow-Release Fertilisers

A 5 wt% sodium alginate solution was prepared by adding 5 g of sodium alginate to 100 mL of deionised water and stirred until entirely dissolved. To this solution was added 5.36 g of ammonium nitrate and 6.73 g of potassium chloride to make up a solution of 5% alginate, 0.669 mM NH_4_NO_3_ and 0.903 mM KCl. The concentrations of NH_4_NO_3_ and KCl used were selected to correspond to concentrations of nitrogen and potassium used in commercial slow-release fertilisers.

A 2 wt% calcium chloride solution was made up, to which was added sufficient ammonium nitrate and potassium chloride such that the final concentrations were 0.669 mM NH_4_NO_3_ and 0.903 mM KCl, to ensure that both the sodium alginate solution and the calcium chloride solution had the same nitrogen and potassium concentrations.

The method outlined in Section 4.2.4 was followed, but the produced slow-release fertilisers beads were not washed with deionized water.

#### 4.2.8. Production of Kelp-Derived Alginate-Based Slow-Release Fertilisers

The same method as outlined in Section 4.2.5 was used to produce kelp-derived slow-release fertilisers; however, instead of using LG alginate, the alginate solution prepared in Section 4.2.3 was used.

#### 4.2.9. Production of Limestone Loaded Hydrogels

A 5 wt% sodium alginate solution was prepared, and 50 g/L limestone (calcium carbonate with an average particle size of 32.49 µm (±0.595)) was added to this solution to produce the limestone added alginate mixture. A 2 wt% calcium chloride solution was made to which also 50 g/L calcium carbonate was added to ensure that no calcium carbonate would be lost in the mixture when the alginate beads would be produced. The fine limestone was kept in suspension through the constant stirring of the solutions. The experimental setup discussed in Section 4.2.4 was used to produce limestone-loaded alginate hydrogel beads. The same method was followed as discussed in Section 4.2.4, but the produced limestone beads were not washed before being dried since this would wash out the nutrients.

#### 4.2.10. Nutrient Release Rates of Slow-Release Fertilisers

Nutrient release tests were conducted to determine the release rate of the fertilizer nutrients entrapped within the alginate hydrogel beads. Briefly, 30 g of the wet slow-release fertiliser beads was added to 300 mL of deionised water and was kept at room temperature. The mixture was stirred at 500 rpm, and 1 mL samples were taken until equilibrium was reached. These samples were appropriately diluted and analysed for potassium, nitrogen, and phosphorus concentrations, using the analytical methods outlined in Section 4.3.2 and Section 4.3.3.

#### 4.2.11. Diffusivity Calculations

The diffusion coefficient indicates a solute’s interaction with a solvent and indicates the rate at which a substance’s mass is transferred through a surface in the diffusion direction into a solvent [75]. The diffusion coefficient is also constant for specific solute and solvent combinations at specific temperatures [76]. Therefore, the diffusion coefficient is used to compare the diffusivity of different solutes in a specific solvent at specific temperatures.

The nutrient release rates of the produced slow-release fertilisers were investigated by measuring the released concentrations of the N and K components over time in deionised water. Equation (3) was derived based on Fick’s Law to determine the diffusion of molecules in ball-shaped matrices [77]:

(3) $$\ln\left(\frac{C_{t}-C_{f}}{C_{0}-C_{f}}\right)=-\frac{\pi^{2}\cdot D}{r^{2}}\cdot t+const$$

In Equation (3), $C_{t}$ is the concentration of the solution at time point t, $C_{0}$ is the initial concentration of the solution at t=0, and $C_{f}$ is the concentration of the solution at a steady state. The converted concentrations were calculated at each time measurement, using Equation (3). The left-hand term of Equation (3) was then plotted against time and a straight line fitted. Equation (3) is linear, and thus it is determined that the gradient of the resulting fitted straight line would be equal to $-\frac{\pi^{2}\cdot D}{r^{2}}$. With the diameter of the slow-release fertiliser beads measured, the diffusion coefficient $\left(D\right)$ of the nutrients in the slow-release fertilisers could be determined.

#### 4.2.12. Solution pH Analysis with the Addition of Produced Hydrogel Beads

A solution was made up by adding 2 g of the dried hydrogel beads and 100 mL deionised water into a 250 mL beaker and then stirred with a magnetic stirrer at a rate of 200 rpm. This was done for both the beads produced in Section 4.2.8 and Section 4.2.9 separately.

The pH of the solution was measured over time using a Hannah HI5522 benchtop multi-meter to determine the release rates of compounds affecting the pH from produced hydrogel beads and thus estimating the release rate of nutrients from the produced hydrogel beads.

### 4.3. Analytical Methods

#### 4.3.1. Phenol-Sulfuric Acid Method

The phenol-sulfuric acid method as outlined in Albalasmeh [78] was used to determine the alginate content of the industrial kelp solid waste samples. Two millilitres of sodium alginate solution was mixed with 1 mL 5 wt% phenol solution in a test tube. Next, 5 mL of concentrated sulfuric acid was added, and the solution was left for 10 min. The solution was then vortexed for 30 s and then placed in a water bath for 20 min at room temperature for colour development. The light absorption of the solution was then analysed at 490 nm on a spectrophotometer. A standard curve was constructed by analysing 0, 0.5, 1 and 2 g/L pure sodium alginate solutions with the same method discussed above. The standard curve was then used as a reference to determine the alginate content of the industrial kelp solid waste samples.

#### 4.3.2. Atomic Absorption Spectroscopy Analysis

Atomic absorption spectroscopy (AAS) was used to determine the potassium concentrations in the samples. A NovAA 400 P machine was used, and acetylene was used as the combustion gas. Three potassium standards (10 ppm, 50 ppm and 100 ppm) were produced by appropriate dilution. A standard curve was developed using these standards and the atomic absorption spectroscope.

The diluted samples collected from the release tests were taken and tested using the AAS machine and converted to concentrations using the developed standard curve.

#### 4.3.3. Spectrophotometry Analysis

The nitrogen concentrations in the diluted samples were determined with the use of Spectroquant^®^ 10–150 mg/L total nitrogen cell tests.

As outlined in the operating procedure of the cell tests, the following procedure was followed: 1 mL of the diluted sample was decanted into an empty glass cell. The sample was then diluted with 9 mL of ultrapure water. One micro-spoonful of Reagent N-1K was added to the cell. The cell was then remixed, and finally, 6 drops of Reagent N-2K were added. The cell was then closed tightly and mixed. The cell was then heat-treated at 120 °C for 1 h in a preheated thermoreactor to initiate the oxidizing reaction. After the hour of heat treatment, the sample was cooled down to room temperature. One millilitre of the cooled sample was then pipetted into a reaction cell provided in the nitrogen (total) cell test kit. One millilitre of the provided Reagent N-3K was added to the reaction cell, and the content was thoroughly mixed. After the content was mixed, the measurement sample was ready for analysis. The sample was then placed into the Spectroquant^®^, and the nitrogen concentration in the sample was recorded.

## Figures and Tables

**Table 1:** Guluronic to mannuronic acid ratio of *Ecklonia maxima* and *Macrocystis pyrifera* from literature [56] and the G/M ratio results of laboratory-grade alginate (LG) and alginate sourced from industrial kelp solid waste (IW).

Alginate Source	G/M Ratio	Reference
IW	0.36	This study
LG	0.53	This study
*Ecklonia maxima*	0.82	[56]
*Macrocystis pyrifera*	0.64	[56]

**Table 2:** Rate parameters (*r*) and the equilibrium swelling ratios (*S_e_*) determined for hydrogels in deionised water at room temperature using the Voigt model. Hydrogels tested were produced using industrial kelp solid waste extracted alginate slurries of varying solid contents (1, 2.5 and 3.33 wt%) and also hydrogels produced from laboratory-grade sodium alginate solutions of varying concentrations (1, 2.5 and 5 wt%). The coefficient of determination (R^2^) indicates how accurately the Voigt model fits the swelling results.

Alginate Source	(wt%)	*r* (s)	*Se* (g/g)	R^2^
Laboratory-grade (LG)	1	1542	0.62	0.972
	2.5	4326	0.71	0.974
	5	7797	0.85	0.983
Industrial kelp solid waste (IW)	1	4047	1.67	0.972
	2.5	5913	1.20	0.985
	3.33	7654	1.31	0.991

**Table 3:** Diffusion coefficients determined of K^+^ and NO_3_^−^ from laboratory-grade (LG) alginate-based and industrial waste extracted alginate (IW) slow-release fertilisers into deionised water at 25 °C. Diffusion coefficients determined by previous research is also shown for comparison.

Fertiliser Nutrient	Alginate Source	Diffusion Coefficient (cm^2^/s)	Reference
Potassium ions	LG	5.02 × 10^−6^	This study
	IW	4.14 × 10^−6^	This study
	K^+^ (in infinite water)	1.96 × 10^−5^	[63]
	PVA membrane	3.91 × 10^−6^	[64]
	Polymer latex	1.86 × 10^−9^	[22]
Nitrate ions	LG	7.07 × 10^−6^	This study
	IW	5.61 × 10^−6^	This study
	NO_3_^−^ (in infinite water)	1.90 × 10^−5^	[63]
	Polymer latex	1.25 × 10^−9^	[22]

## Data Availability

Data are available as part of a postgraduate thesis, which can be found at http://hdl.handle.net/10019.1/124848 (accessed on 28 July 2022).

## References

[1] Huho J.M., Ngaira J.K.W., Ogindo H.O., Masayi N. (2012). The changing rainfall pattern and the associated impacts on subsistence agriculture in Laikipia East District, Kenya. J. Geogr. Reg. Plan..

[2] Du Plessis J.A., Burger G.J. (2015). Investigation into increasing short-duration rainfall intensities in South Africa. Water SA.

[3] Diacono M., Montemurro F. (2010). Long-term effects of organic amendments on soil fertility. A review. Agron. Sustain. Dev..

[4] Bogard M.J., Donald D.B., Finlay K., Leavitt P.R. (2012). Distribution and regulation of urea in lakes of central North America. Freshwat. Biol..

[5] Zafari A., Kianmehr M.H. (2012). Management and reduction of chemical nitrogen consumption in agriculture. Am. J. Plant Sci..

[6] Ni B., Liu M., Lü S., Xie L., Wang Y. (2011). Environmentally friendly slow-release nitrogen fertilizer. J. Agric. Food Chem..

[7] Rashidzadeh A., Olad A. (2014). Slow-released NPK fertilizer encapsulated by NaAlg-g-poly(AA-co-AAm)/MMT superabsorbent nanocomposite. Carbohydr. Polym..

[8] Orikiriza L.J.B., Agaba H., Tweheyo M., Eilu G., Kabasa J.D., Hüttermann A. (2009). Amending soils with hydrogels increases the biomass of nine tree species under non-water stress conditions. Clean Soil Air Water.

[9] Davidson D.W., Verma M.S., Gu F.X. (2013). Controlled root targeted delivery of fertilizer using an ionically crosslinked carboxymethyl cellulose hydrogel matrix. SpringerPlus.

[10] Oshunsanya S.O., Oshunsanya S.O. (2019). Introductory Chapter: Relevance of Soil pH to Agriculture. Soil pH for Nutrient Availability and Crop Performance.

[11] Schwaeble C.F., Pott R.W.M., Goosen N.J. (2021). The effect of sodium alginate, lignosulfonate and bentonite binders on agglomeration performance and mechanical strength of micro-fine agricultural lime pellets. Part. Sci. Technol..

[12] Goulding K.W.T. (2016). Soil acidification and the importance of liming agricultural soils with particular reference to the United Kingdom. Soil Use Manag..

[13] Chang L., Xu L., Liu Y., Qui D. (2021). Superabsorbent polymers used for agricultural water retention. Polym. Test..

[14] Elshafie H.S., Camele I. (2021). Applications of absorbent polymers for sustainable plant protection and crop yield. Sustainability.

[15] Seetapan N., Wongsawaeng J., Kiatkamjornwong S. (2011). Gel strength and swelling of acrylamide-protic acid superabsorbent copolymers. Polym. Adv. Technol..

[16] Sharma R., Bajpai J., Bajpai A.K., Acharya S., Shrivastava R.B., Shukla S.K. (2014). Designing slow water-releasing alginate nanoreserviors for sustained irrigation in scanty rainfall areas. Carbohydr. Polym..

[17] Goldhamer D.A., Fereres E., Mata M., Girona J., Cohen M. (1999). Sensitivity of Continuous and Discrete Plant and Soil Water Status Monitoring in Peach Trees Subjected to Deficit Irrigation. J. Am. Soc. Hortic. Sci..

[18] Flanagan D.C., Chaudhari K., Norton L.D. (2002). Polyacrylamide soil amendment effects on runoff and sediment yield on steep slopes: PART II. natural rainfall conditions. Trans. Am. Soc. Agric. Eng..

[19] Zohuriaan-Mehr M.J., Omidian H., Doroudiani S., Kabiri K. (2010). Advances in non-hygienic applications of superabsorbent hydrogel materials. J. Mater. Sci..

[20] Madduma-Bandarage U.S.K., Madihally S.V. (2020). Synthetic hydrogels: Synthesis, novel trends, and applications. J. Appl. Polym. Sci..

[21] Sofyane A., Ablouh E., Lahcini M., Elmeziane A., Khouloud M., Kaddami H., Raihane M. (2020). Slow-release fertilizers based on starch acetate/glycerol/polyvinyl alcohol biocomposites for sustained nutrient release. Mater. Today Proc..

[22] An D., Yang L., Liu B., Wang T.J., Kan C. (2017). Diffusion performance of fertilizer nutrient through polymer latex film. J. Agric. Food Chem..

[23] Achmon Y., Dowdy F.R., Simmons C.W., Zohar-Perez C., Rabinovitz Z., Nussinovitch A. (2019). Degradation and bioavailability of dried alginate hydrocolloid capsules in simulated soil system. J. Appl. Polym. Sci..

[24] Hurtado A., Aljabali A.A.A., Mishra V., Tambuwala M.M., Serrano-Aroca Á. (2022). Alginate: Enhancement strategies for advanced applications. Int. J. Mol. Sci..

[25] Hecht H., Srebnik S. (2016). Structural characterization of sodium alginate and calcium alginate. Biomacromolecules.

[26] Tønnesen H.H., Karlsen J. (2002). Alginate in drug delivery systems. Drug Dev. Ind. Pharm..

[27] Imeson A. (1992). Thickening and Gelling Agents for Food.

[28] Gao J., Li Z., Wang Z., Chen T., Hu G., Zhao Y., Han X. (2022). Facile synthesis of sustainable tannin/sodium alginate composite hydrogel beads for efficient removal of methylene blue. Gels.

[29] Zactiti E.M., Kieckbusch T.G. (2009). Release of potassium sorbate from active films of sodium alginate crosslinked with calcium chloride. Packag. Technol. Sci..

[30] Davidovich-Pinhas M., Bianco-Peled H. (2010). A quantitative analysis of alginate swelling. Carbohydr. Polym..

[31] Gallagher L., Smith A., Kavanagh K., Devereux M., Colleran J., Breslin C., Richards K., McCann M., Rooney A.D. (2021). Preparation and antimicrobial properties of alginate and serum albumin/glutaraldehyde hydrogels impregnated with silver(I) ions. Chemistry.

[32] Abd El-Rehim H.A. (2006). Characterization and possible agricultural application of polyacrylamide/sodium alginate crosslinked hydrogels prepared by ionizing radiation. J. Appl. Polym. Sci..

[33] Shivakumara L.R., Demappa T. (2019). Synthesis and swelling behavior of sodium alginate/poly(Vinyl alcohol) hydrogels. Turk. J. Pharm. Sci..

[34] Kartik A., Akhil D., Lakshmi D., Gopinath K.P., Arun J., Sivaramakrishnan R., Pugazhendhi A. (2021). A critical review on production of biopolymers from algae biomass and their applications. Bioresour. Technol..

[35] Mazen A.M., Radwan D.E.M., Ahmed A.F. (2015). Growth responses of maize plants cultivated in sandy soil amended by different superabsorbant hydrogels. J. Plant Nutr..

[36] Idrissi A.E., Gharrak A.E., Achagri G., Essamlali Y., Amadine O., Akil A., Sair S., Zahouil M. (2022). Synthesis of urea-containing sodium alginate-g-poly(acrylic acid-co-acrylamide) superabsorbent-fertilizer hydrogel reinforced with carboxylated cellulose nanocrystals for efficient water and nitrogen utilization. J. Environ. Chem. Eng..

[37] Michalak I., Chojnacka K. (2013). Algal compost—Toward sustainable fertilization. Rev. Inorg. Chem..

[38] Sharma H.S.S., Fleming C., Selby C., Rao R.J., Martin T. (2014). Plant biostimulants: A review on the processing of macroalgae and use of extracts for crop management to reduce abiotic and biotic stresses. J. Appl. Phycol..

[39] Arioli T., Mattner S.W., Winberg P.C. (2015). Applications of seaweed extracts in Australian agriculture: Past, present and future. J. Appl. Phycol..

[40] Lötze E., Hoffman E.W. (2016). Nutrient composition and content of various biological active compounds of three South African-based commercial seaweed biostimulants. J. Appl. Phycol..

[41] Fatehi H., Ong D.E.L., Yu J., Chang I. (2021). Biopolymers as green binders for soil improvement in geotechnical applications: A review. Geosciences.

[42] Pérez-Hernández H., Fernández-Luqueño F., Huerta-Lwanga E., Mendoza-Vega J., José D.Á.-S. (2020). Effect of engineered nanoparticles on soil biota: Do they improve the soil quality and crop production or jeopardize them?. Land Degrad. Dev..

[43] Bouranis D.L., Theodoropoulos A.G., Drossopoulos J.B. (1995). 1995. Designing synthetic polymers as soil conditioners. Commun. Soil Sci. Plant Anal..

[44] Hüttermann A., Zommorodi M., Reise K. (1999). Addition of hydrogels to soil for prolonging the survival of *Pinus halepensis* seedlings subjected to drought. Soil Tillage Res..

[45] Campos E.V.R., De Oliveira J.L., Fraceto L.F., Singh B. (2014). Polysaccharides as safer release systems for agrochemicals. Agron. Sustain. Dev..

[46] Lackey B.A., Muldoon A.E., Jaffee B.A. (1993). Alginate pellet formulation of hirsutella rhossiliensis for biological control of plant-parasitic nematodes. Biol. Control.

[47] Ekanayake S.A., Godakumbura P.I. (2021). Synthesis of a dual-functional nanofertilizer by embedding ZnO and CuO nanoparticles on an alginate-based hydrogel. ACS Omega.

[48] Knijnenburg J.T.N., Kasemsiri P., Amornrantanaworn K., Suwanree S., Iamamornphan W., Chindaprasirt P., Jetsrisuparb K. (2021). Entrapment of nano-ZnO into alginate/polyvinyl alcohol beads with different crosslinking ions for fertilizer applications. Int. J. Biol. Macromol..

[49] Liu S., Wu Q., Sun X., Yue Y., Tubana B., Yang R., Cheng H.N. (2021). Novel alginate-cellulose nanofiber-poly(vinyl alcohol) hydrogels for carrying and delivering nitrogen, phosphorus and potassium chemicals. Int. J. Biol. Macromol..

[50] Di Martino A., Khan Y.A., Durpekova S., Sedlarik V., Elich O., Cechmankova J. (2021). Ecofriendly renewable hydrogels based on whey protein and for slow release of fertilizers and soil conditioning. J. Clean. Prod..

[51] Arafa E.G., Sabaa M.W., Mohamed R.R., Elzanaty A.M., Abdel-Gawad O.F. (2022). Preparation of biodegradable sodium alginate/carboxymethylchitosan hydrogels for the slow-release of urea fertilizer and their antimicrobial activity. React. Funct. Polym..

[52] Van der Merwe R.D.T. (2022). Macroalgal-Derived Alginate Soil Amendments for Water Retention, Reduced Nutrient Release Rate, and Soil pH Control.

[53] McHugh D.J. (2003). A Guide to the Seaweed Industry.

[54] Zhao Y., Chen H.H., Wang Y.S., Li Q.Q. (2016). Effect of sodium alginate and its guluronic acid/mannuronic acid ratio on the physicochemical properties of high-amylose cornstarch. Starch Staerke.

[55] Zhang H., Cheng J., Ao Q. (2021). Preparation of alginate-based biomaterials and their applications in biomedicine. Mar. Drugs.

[56] Skjåk-Bræk G., Draget K.I., Matyjaszewski K., Möller M. (2012). Alginates: Properties and Applications. Polymer Science: A Comprehensive Reference.

[57] Devi G.K., Kumar P.S., Kumar K.S. (2016). Green synthesis of novel silver nanocomposite hydrogel based on sodium alginate as an efficient biosorbent for the dye wastewater treatment: Prediction of isotherm and kinetic parameters. Desalination Water Treat..

[58] Serrano-Aroca Á., Ruiz-Pividal J.F., Llorens-Gámez M. (2017). Enhancement of water diffusion and compression performance of crosslinked alginate films with a minuscule amount of graphene oxide. Sci. Rep..

[59] Zhang K., Feng W., Jin C. (2020). Protocol efficiently measuring the swelling rate of hydrogels. MethodsX.

[60] Kumar S., Sahoo D. (2017). A comprehensive analysis of alginate content and biochemical composition of leftover pulp from brown seaweed *Sargassum wightii*. Algal Res..

[61] Llive L.M., Perullini M., Santagapita P.R., Schneider-teixeira A., Deladino L. (2020). Controlled release of fertilizers from Ca (II)-alginate matrix modified by yerba mate (*Ilex paraguariensis*) waste. Eur. Polym. J..

[62] Mandal S., Kumar S.S., Krishnamoorthy B., Basu S.K. (2010). Development and evaluation of calcium alginate beads prepared by sequential and simultaneous methods. Braz. J. Pharm. Sci..

[63] Lide D.R., Baysinger G., Berger L.I., Goldberg R.N., Kehiaian H.V., Kuchitsu K., Rosenblatt G., Roth D.L., Zwillinger D. (2007). CRC Handbook of Chemistry and Physics.

[64] Papancea A., Patachia S. (2014). Electrolytes diffusion through modified PVA hydrogel membrane. Bull. Transilv. Univ. Bras..

[65] Fageria N.K., Nascente A.S., Sparks D.S. (2014). Chapter Six—Management of Soil Acidity of South American Soils for Sustainable Crop Production. Advances in Agronomy.

[66] Schwab G., Murdock L.W., Ditsch D., Rasnake M., Sikora F.J., Frye W. (2007). Agricultural Lime Recommendations Based on Lime Quality.

[67] Gomez C.G., Lambrecht M.V.P., Lozano J.E., Rinaudo M., Villar M.A. (2009). Influence of the extraction-purification conditions on final properties of alginates obtained from brown algae (*Macrocystis pyrifera*). Int. J. Biol. Macromol..

[68] Dubois M., Gilles K.A., Hamilton J.K., Rebers P.A., Smith F. (1956). Colorimetric method for determination of sugars and related substances. Anal. Chem..

[69] Venkatesan J., Lowe B., Anil S., Manivasagan P., Kheraif A.A.A., Kang K.H., Kim S.K. (2015). Seaweed polysaccharides and their potential biomedical applications. Starch/Staerke.

[70] Sluiter A., Hames B., Ruiz R., Scarlata C., Sluiter J., Templeton D. (2008). Determination of Sugars, Byproducts, and Degradation Products in Liquid Fraction Process Samples—NREL/TP-510-42623.

[71] Wang W., Chen F., Wang Y., Wang L., Fu H., Zheng F., Beecher L. (2018). Optimization of reactions between reducing sugars and 1-phenyl-3-methyl-5-pyrazolone (PMP) by response surface methodology. Food Chem..

[72] Rashidzadeh A., Olad A., Reyhanitabar A. (2015). Hydrogel/clinoptilolite nanocomposite-coated fertilizer: Swelling, water-retention and slow-release fertilizer properties. Polym. Bull..

[73] Chen Y.C., Chen Y.H. (2019). Thermo and pH-responsive methylcellulose and hydroxypropyl hydrogels containing K2SO4 for water retention and a controlled-release water-soluble fertilizer. Sci. Total Environ..

[74] Lejcuś K., Śpitalniak M., Dabrowska J. (2018). Swelling behaviour of superabsorbent polymers for soil amendment under different loads. Polymers.

[75] Mostinsky I.L. (2008). Diffusion Coefficient. A-to-Z Guide to Thermodynamics, Heat and Mass Transfer, and Fluids Engineering.

[76] Daintith J., Daintith J. (2008). A Dictionary of Chemistry.

[77] Grunwald P. (1989). Determination of effective diffusion coefficients—An important parameters for the efficiency of immobilized biocatalysts. Biochemical Education. Biochem. Educ..

[78] Albalasmeh A.A., Berhe A.A., Ghezzehei T.A. (2013). A new method for rapid determination of carbohydrate and total carbon concentrations using UV spectrophotometry. Carbohydr. Polym..

